# Efficiency of Single Dose of Tolvaptan Treatment During the Triphasic Episode After Surgery for Craniopharyngioma

**DOI:** 10.4274/jcrpe.galenos.2018.2018.0182

**Published:** 2019-05-28

**Authors:** Fatih Gürbüz, Mehmet Taştan, İhsan Turan, Bilgin Yüksel

**Affiliations:** 1 Çukurova University Faculty of Medicine, Department of Pediatric Endocrinology, Adana, Turkey

**Keywords:** Inappropriate antidiuretic hormone syndrome, tolvaptan, children

## Abstract

Inappropriate antidiuretic hormone syndrome (SIADH) may develop after intracranial surgery. SIADH in the pediatric age group is usually encountered in patients with an intracranial mass both before and after surgery. Fluid restriction is the standard therapy in SIADH. However, a resistant, hyponatremic pattern may be encountered in some cases. Vaptans have been recently introduced for treatment of hyponatremia due to SIADH. There is inadequate data concerning tolvaptan treatment in pediatric patients. We present a 13 year-old female with SIADH of triphasic episode who was transferred to our clinic after surgery for craniopharyngioma. Resistant hyponatremia did not resolve despite fluid restriction and hypertonic saline support. The patient responded rapidly to a single dose of tolvaptan, with no adverse effect, which resulted in successful control of her SIADH.

**What is already known on this topic?**

Hyponatremia in patients with inappropriate antidiuretic hormone (ADH) syndrome (SIADH) is caused by the combination of excess ADH-induced water retention and secondary solute loss. Vaptans, arginine-vasopressin receptor antagonists, are an alternative for use in SIADH in adults. In children, vaptan treatment has not been approved for SIADH.

**What this study adds?**

We report successful tolvaptan treatment in a child with SIADH. Furthermore, we report that only one dose of tolvaptan in triphasic episode was effective.

## Introduction

The syndrome of inappropriate antidiuretic hormone (ADH) secretion (SIADH) is a disorder of impaired water excretion caused by the inability to suppress secretion of ADH (1). SIADH is clinically serious and one of the causes of hyponatremia in hospitalized patients (2). The etiology of SIADH involves excess ADH production due to cranial surgery, malignancies, meningitis-encephalitis, hemorrhage, other cerebral pathologies, pulmonary malignancies and drugs (2,3).

In clinical practice, the gold standard approach to SIADH is fluid restriction and it is widely used (4,5,6). In more severe forms, with neurological symptoms, administration of hyperosmolar saline combined with furosemide may be required. Additionally, the underlying etiology should be treated if possible (3,5,6,7,8,9,10). However, even these treatments may be inadequate for some patients with SIADH.

In the last decade, a new alternative treatment for SIADH, vaptans which are arginine-vasopressin receptor antagonists, has become available (8). Vaptans, act by preventing the insertion of aquaporin 2 water channels into the apical membrane, promoting reabsorption of water and resulting in excretion of diluted urine (3,5,11).

Tolvaptan is one of the vaptans and is a selective V2 receptor antagonist, whereas conivaptan is a non-selective V1/V2 receptor antagonist (3,5,6,10). Conivaptan is approved by the United States Food and Drug Administration (FDA) for hypervolemic (nephrotic syndrome, cardiac failure and cirrhosis) or euvolemic hyponatremia (SIADH) treatment in adults, but not in children (10). Peters et al (10) described the first successful treatment with conivaptan in a pediatric refractory SIADH patient. It was also reported that conivaptan played a key role in the management of their case with SIADH and that no adverse effects had developed (10).

Tolvaptan has been approved by the FDA for adults since 2009 and has been successfully used in the treatment of hyponatremia due to SIADH and autosomal dominant polycystic kidney disease (1,9,11,12). However data on the safety, efficacy and optimal dose of tolvaptan in pediatric patients are limited. There are a few case reports concerning tolvaptan therapy for pediatric SIADH (3,5,6,13). Similarly, there is little evidence of the use of tolvaptan in the treatment of pediatric hypervolemic hyponatremia, such as that observed in cardiac failure and nephrotic syndrome (14,15,16,17). Successful tolvaptan treatment has been reported in three children with SIADH (ROHHAD syndrome, large sellar-suprasellar tumor and surgery of astrocytoma) (5), in a patient with intracranial lymphoma (3) and in a child with nephrotic syndrome (17). In addition 28 pediatric cases with cardiac failure, treated with tolvaptan have been reported (15). All these case series reported that tolvaptan therapy was effective, safe and well tolerated in hyponatremic children.

Here, we report the first pediatric case of severe and symptomatic hyponatremia due to SIADH, successfully treated with single dose tolvaptan in Turkey.

## Case Report

A 13-year-old girl with a 3-week history of headache and reduction in vision was referred to our practice because of possible endocrine problems due to craniopharyngioma. She was the third child of non-related parents. Her birth history was unremarkable. Her height was 150.8 cm [-1.19 standard deviation (SD)] and her weight was 60.2 kg (1.23 SD). Physical examination was normal except for right eye exotropia and accompanying reduction in vision.

**Figure 1:**
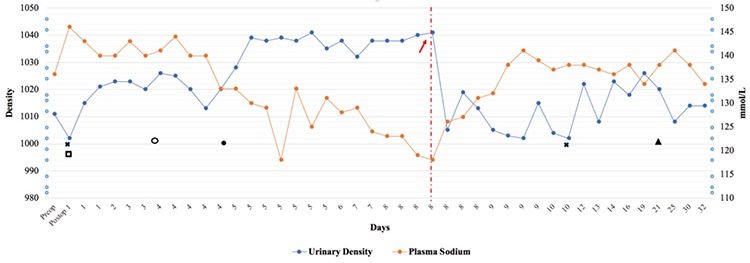
Plasma sodium and urinary output of the patient. Therapy is indicated by the following symbols on the chart: Cross-initiation of desmopressin; open square-initiation of dexamethasone; open circle-initiation of levothyroxine; point-desmopressin cessation; arrow-tolvaptan treatment; triangle-switching from dexamethasone to hydrocortisone

No endocrine abnormalities were detected before the craniopharyngioma operation (see Table 1). On the first postoperative day, dexamethasone treatment for brain-associated surgery was started by the neurosurgeon. Therefore no additional steroid treatment was given in case of central adrenal insufficiency. Furthermore, the patient was polyuric (5.6 mL/kg/h), plasma sodium was 146 mmol/L (reference range 135-145), plasma osmolality was 303 mOsm/kgH_2_O and urinary density was 1002. Desmopressin acetate (0.1 µg/kg/day, melt form) treatment was started for diabetes insipidus (DI). Desmopressin treatment improved her polyuria and plasma sodium concentration. On the fourth postoperative day, levothyroxine (100 µg/day) replacement therapy was started for central hypothyroidism. The patient had also developed hyponatremia, starting on postoperative day four, which gradually worsened. On the fifth postoperative day, urinary output of the patient decreased to 0.7 mL/kg/h. Evaluation of laboratory findings (plasma sodium 128 mmol/L, plasma osmolality 267 mOsm/kgH_2_O, urinary density 1039) led to the diagnosis of SIADH. Plasma copeptin/ADH levels could not be measured. The findings suggested that SIADH was the second stage of the triphasic condition encountered after cranial surgery. Initial management included fluid restriction (administered fluid: total 800 mL/m^2^/day) and cessation of desmopressin treatment. Despite fluid restriction for four days, the patient’s blood sodium levels continued to decreas, to 118 mmol/L, and urine density was 1039. Hypertonic saline therapy (3% saline to raise the serum sodium by 10 mEq/L) was also added due to persistence of hyponatremia. However, SIADH could not be controlled and severe hyponatremia continued. In addition, the patient’s condition began to worsen and mild loss of consciousness occurred. Therefore, it was decided to start low-dose oral tolvaptan treatment (0.13 mg/kg/day) on the eighth postoperative day. A written consent form was obtained from the parents for the use of tolvaptan. One hour after oral intake of Tolvaptan, the urine output and plasma sodium levels of the patient began to correct. Urinary output increased to 8.1 mL/kg/h, urinary density reduced to 1001. One dose of tolvaptan administered to the patient was sufficient to control SIADH and no further treatment was given. Moreover, desmopressin treatment was restarted because of the development of DI 42 hours after the administration of tolvaptan (plasma sodium 138 mmol/L, plasma osmolality 296 mOsm/kgH_2_O, urinary output 6.6 mL/kg/h and urinary density 1002). The patient has had persistent DI on follow up which has required desmopressin therapy (Figure 1).

## Discussion

Here we report successful tolvaptan administration in a patient who developed severe and uncontrolled hyponatremia due to SIADH. To our knowledge, this is the first report from Turkey of successful pediatric tolvaptan treatment.

Hypothalamus and/or tract damage due to neurosurgery or trauma may frequently result in a typical triphasic response (18,19,20). First, transient DI develops, beginning within 24 hours and lasting from four to five days. The DI is due to reflex inhibition of ADH release because of hypothalamic dysfunction. Following that, on days 6-10, a transient SIADH develops, caused by release of stored ADH from the disrupted posterior pituitary. Finally, DI reoccurs, after the posterior pituitary ADH stores are consumed. This third phase DI may be permanent or transient (19,20). Our patient exhibited this triphasic response.

There are two different approaches to managing central DI in patients who have undergone cranial surgery. The first approach is to employ fluids and avoid the use of vasopressin. This approach may be particularly useful for managing acute postoperative DI in young children. Vasopressin therapy may mask the emergence of the second SIAD phase of the triple phase neurohypophyseal response to neurosurgical injury (21). The other approach is treatment with vasopressin. Our patient was 13-years-old with a weight of 60.2 kg which is the same weight as some adults. For this reason, we preferred to use vasopressin for the DI, and did not observe the masking of the emergence of the SIAD when DI was thought to persist transiently for four or five days (19,20).

Also, a surgery of longer duration has been associated with developing the triphasic response (22). Our patient’s surgery took about 10.5 hours. However, in the second transient SIADH phase, the patient developed severe and uncontrollable, symptomatic hyponatremia. For this reason, even though it was probable that this second phase was temporary, intervention was unavoidable because of the severe hyponatremia together with resistance to fluid restriction and hypertonic saline therapy. Vaptans were the most appropriate choice as an alternative treatment. We preferred to use tolvaptan in this case, which is a selective V2 receptor antagonist, as it may be more effective and cause fewer side effects. Her resistant hyponatremia improved dramatically and rapidly following a single low-dose tolvaptan administration.

Marx-Berger et al (6) reported the use of tolvaptan treatment in two infants with non-improving hyponatremia due to SIADH. The treatment was initiated at a dose of 0.8 mg/kg/day, and the dose was reduced to 0.22 mg/kg/day upon the onset of hypernatremia on the second day of treatment in one infant. Tolvaptan was used for seven months in one of the infants and for 13 months in the other, without any problems. In our patient, a single dose of tolvaptan at a dose of 0.13 mg/kg/day was sufficient, and in our patient, even desmopressin was started due to the development of hypernatremia and conversion of the condition to DI. In another article, tolvaptan treatment was given to three SIADH patients aged between four and seven years, at a dose of 0.05-0.3 mg/kg/day, and low dose tolvaptan treatment was continued for as long as 3-4 years without complications (5). Long-term therapy such as this can be given with confidence as the reported incidence of adverse effects is extremely low.

However, although the SIADH in our patient was very likely temporary and would return to DI, the final phase, spontaneously, her clinical condition was too severe to wait. Therefore, we accelerated the passage to the third phase with tolvaptan therapy. Finally, 42 hours after tolvaptan, desmopressin treatment for DI was started. No side effects were observed in our patient which could be attributed to tolvaptan.

For tolvaptan, time of onset of action for aquaretic and sodium increasing effects is two to four hours with a peak effect at between four and eight hours (23). Willemsen et al (3) suggested starting at a low dose to avoid rapid correction of hyponatremia. Furthermore, Peters et al (10) noted the correction of hyponatremia on the first day after conivaptan was given. In our patient, the aqauretic effect of tolvaptan began after only one hour following ingestion, despite the low dose. This was a fast and immediate effect after a single dose tolvaptan treatment. Therefore, patients need to be closely followed from the first hour in terms of both urine output and increase in serum sodium levels.

The use of tolvaptan or conivaptan therapy in childhood has still not been approved by FDA or the European Medicines Agency (EMA). The most important reason for this is that there is not enough clinical experience in terms of safety and effectiveness in children. We observed a successful treatment result with tolvaptan in a pediatric patient who suffered from SIADH. We report that only one low dose of tolvaptan in the triphasic episode was remarkably effective in correcting a serious clinical situation. This report of vaptan use, together with future cases, will increase the clinical evidence base in order for the FDA and EMA to decide the licensing status of this type of therapy.

## Figures and Tables

**Table 1:**
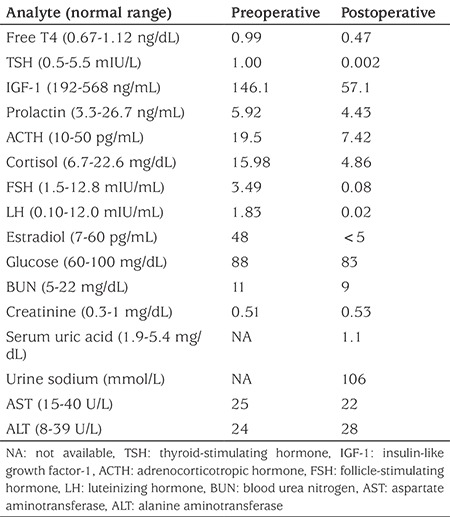
Laboratory findings of the patient
